# Anti-fibrotic effect of *Spirulina maxima*-derived extracellular vesicles: possible role of PARK7 and HSP70 chaperones

**DOI:** 10.3389/fbioe.2026.1761403

**Published:** 2026-03-31

**Authors:** Beáta Szebeni, Mária Bernáth, Péter Bokrossy, Domonkos Pap, Zoltán Molnár, Tamás Mészáros, Dorina Lenzinger, Tamás Visnovitz, Marcell Pálmai, Judith Mihály, Zoltán Varga, Nóra Fekete, Éva Pállinger, Apor Veres-Székely, Csenge Szász, Edit Buzás, Attila J. Szabó, Ádám Vannay

**Affiliations:** 1 Pediatric Center, MTA Center of Excellence, Semmelweis University, Budapest, Hungary; 2 HUN-REN-SU Pediatrics and Nephrology Research Group, Budapest, Hungary; 3 Department of Plant Sciences, Albert Kázmér Faculty of Agricultural and Food Sciences in Mosonmagyaróvár, Széchenyi István University, Mosonmagyaróvár, Hungary; 4 Department of Translational Medicine, Nanomedicine Research and Education Center, Semmelweis University, Budapest, Hungary; 5 SeroScience Ltd., Budapest, Hungary; 6 Heart and Vascular Center, Faculty of Medicine, Semmelweis University, Budapest, Hungary; 7 Institute of Genetics, Cell - and Immunobiology, Semmelweis University, Budapest, Hungary; 8 Department of Plant Physiology and Molecular Plant Biology, ELTE Eötvös Loránd University, Budapest, Hungary; 9 Biological Nanochemistry Research Group, Institute of Materials and Environmental Chemistry, HUN-REN Research Centre for Natural Sciences, Budapest, Hungary; 10 Department of Physical Chemistry and Materials Science, Faculty of Chemical Technology and Biotechnology, Budapest University of Technology and Economics, Budapest, Hungary; 11 Department of Biophysics and Radiation Biology - HUN-REN Office for Supported Research Groups, Faculty of Medicine, Semmelweis University, Budapest, Hungary; 12 HUN-REN-SU Translational Extracellular Vesicle Research Group, Budapest, Hungary

**Keywords:** extracellular vesicle, peritoneal fibroblasts, peritoneal fibrosis, peritoneal mesothelial cells, size exclusion chromatography, *Spirulina maxima* algae, ultrafiltration

## Abstract

**Introduction:**

*Spirulina maxima* (Sm), a blue-green microalgae, is well known for its rich nutritional composition, antioxidant, and anti-inflammatory properties. In this study, we found that small extracellular vesicles (sEVs) isolated from *Sm* exhibit antifibrotic activity.

**Methods:**

*Sm* derived sEVs (*Sm*_sEV) were purified from the Sm culture medium using tangential flow filtration (TFF), followed by size-exclusion chromatography (SEC). Characterization of the sEVs was performed using nanoparticle tracking analysis (NTA), transmission electron microscopy (TEM), immunogold TEM, surface-enhanced IR spectroscopy (SEIRS), and flow cytometry (FC). Internalization of DiI-labeled sEVs by human primary peritoneal mesothelial cells (P-MCs) and peritoneal fibroblasts (p-FBs) was examined using fluorescence microscopy. The effect of sEVs on mesenchymal transition of P-MCs and activation of P-FBs was investigated by immunofluorescent staining, MTT cell-proliferation, and Sirius Red collagen accumulation assays, respectively. The antifibrotic relevance of *Sm*_sEV was further investigated in a chlorhexidine digluconate (CG)-induced mouse model of peritoneal fibrosis.

**Results:**

The isolated *Sm*_sEV exhibited a spherical morphology, with a size range of 150.0 ± 67.6 nm, and the protein-to-lipid ratio (P/L_spectr) was 2.27 ± 0.07. The sEVs cargo contained Parkinson’s disease protein 7 (PARK7), and heat shock protein 70 (HSP70). DiI-labeled sEVs were successfully internalized by both P-MCs and P-FBs and inhibited TGF-β-induced mesenchymal transition in P-MCs and the collagen production and PDGF-B-induced proliferation in P-FBs. *In vivo*, intraperitoneal administration of *Sm*_sEVs reduced CG-induced submesothelial thickening, fibronectin, and collagen type I alpha 1 immunopositivity, and increased cytokeratin 18, immunopositivity in the mesothelial layer.

**Discussion:**

These findings highlight the antifibrotic effect of *Sm*_sEV and support their further investigation in the context of fibrosis.

## 1 Introduction

Peritoneal fibrosis is a progressive pathological process characterized by excessive extracellular matrix (ECM) deposition and submesothelial thickening of the peritoneal membrane, resulting from osmotic stress, glucose degradation products in the dialysis solution, and recurrent peritonitis during long-term peritoneal dialysis (PD) (Ito et al., 2024; Terri et al., 2021). The architectural changes of the peritoneum limit the effectiveness of PD, necessitating a switch to another modality of renal replacement therapy or to renal transplantation. In 0.5%–4.4% of cases, life-threatening encapsulating peritoneal sclerosis (EPS) develop (Petri et al., 2016; Jagirdar et al., 2019). Despite recent advances in understanding its pathogenesis, effective therapeutic strategies to prevent or reverse peritoneal fibrosis remain limited.

In recent years, EVs particularly those derived from mesenchymal stem cell (MSC) have emerged as key mediators of intercellular communication, capable of modulating inflammatory, and regenerative pathways (Zhang et al., 2020; Roszkowski, 2024; Berumen et al., 2021). However, the clinical application of MSC-derived EVs is limited by several factors, including regulatory concerns regarding their heterogeneity (Shen et al., 2025), stability (Fuloria et al., 2021), incompletely understood mechanisms of action (Fuloria et al., 2021; Yadav et al., 2024) and the high cost of their production (Silva et al., 2025; Ulpiano et al., 2023). Recently, EVs derived from non-mammalian sources, including microalgae, are gaining attention due their unique bioactive properties (Adamo et al., 2021; Garaeva et al., 2025), therapeutic potential (Garaeva et al., 2025), and importantly their low production cost (Paterna et al., 2022).

Algae are ubiquitous in the environment and occur wherever moisture and sufficient light are available, including freshwater and marine ecosystems. Therefore, it is not surprising that immune tolerance toward algal components has evolved in humans. Accordingly, algae constitute an integral part of the human diet, primarily through their use as food additives, including gelling agents, thickening agents, emulsifiers, as well as natural colorants (Tavares et al., 2023).

Recent publications also revealed that algal or microalgal EVs exhibit good biocompatibility and low toxicity in mammalian systems (Adamo et al., 2024; Bayat et al., 2021; Brizzi et al., 2025). Indeed, Garaeva and coworkers demonstrated that EVs derived from *Chlamydomonas reinhardtii* and *Parachlorella kessleri* can deliver cargo to human cell lines without cytotoxic effects, supporting their potential use as biomedical delivery systems (Garaeva et al., 2025). EVs of *Tetraselmis chuii origin* showed no cytotoxic effects *in vitro*, and they exhibited anti-inflammatory properties (Adamo et al., 2024). Following intravenous administration, these EVs were shown to be biocompatible in mice, with no signs of acute toxicity, including changes in body weight, evidence of liver or kidney damage, or signs of immune activation. However, comprehensive long-term toxicity studies and repeated dose assessments in mammalian models remain limited or are still ongoing.

*Spirulina sp*, a filamentous cyanobacterium long recognized for its rich content of antioxidants (Finamore et al., 2017), anti-inflammatory compounds (Finamore et al., 2017; Wu et al., 2016), and bioactive peptides (Montalvo et al., 2020), has been investigated for a variety of health benefits (Deng and Chow, 2010). Previously, although dietary *Spirulina platensis* had no effect on liver fibrosis, it was shown to reduce pro-inflammatory responses in the splenocytes in a high-fat diet model (Pham et al., 2019). In another study, treatment with a liquid extract of Spirulina platensis significantly reduced hepatic fibrosis and decreased the expression of key pro-fibrotic genes, along with reducing inflammation and oxidative stress, in a Western diet–induced non-alcoholic steatohepatitis mouse model (Coué et al., 2019). These observations suggest that *S. platensis* exert antioxidant and immunomodulatory properties and may also have anti-fibrotic effects. However, their role in classical fibrosis models remains completely unclear.

Although the successful uptake of algal EVs by human cells and their anti-inflammatory, antioxidant, and antimicrobial activities have been demonstrated (Adamo et al., 2024; Bayat et al., 2021; Brizzi et al., 2025), to the best of our knowledge, this is the first study investigating the role of algal EVs in fibrotic processes. In this study, therefore we isolated and characterized *Sm*_sEV and investigated their antifibrotic effects in a mouse model of peritoneal fibrosis. We aimed to evaluate whether *Sm*_sEV can modulate key cellular and molecular mechanisms of fibrosis, such as proliferation, mesenchymal transition and ECM accumulation of peritoneal mesothelial cells and fibroblasts, respectively.

## 2 Methods

### 2.1 Algae cultivation

The MACC-909 *Sm* strain was obtained from the Mosonmagyaróvár Algae Culture Collection (MACC). The strain was cultivated in 250 mL Erlenmeyer flasks containing tap water-based Spirulina medium, as kindly provided by Zoltán Molnár. Cultures were maintained on an orbital shaker at 150 rpm at room temperature (∼22 °C–25 °C). Optimal growth conditions included a 12-h light/12-h dark photoperiod using a standard white light bulb. The filamentous morphology of the MACC-909 *Sm* strain were examined using light microscopy with an Olympus IX-81 fluorescence microscope system (Olympus Corporation, Japan).

### 2.2 Extracellular vesicle purification from *Spirulina maxima*

To remove microbiological contaminants and cell debris, *Sm* culture was centrifuged at 160 × g for 20 min (Rotanta 460R, Hettich Zentrifugen, Germany). The resulting supernatant was then filtered through 0.22 µm pore size filter (Millipore® Steritop® Vacuum Bottle Top Filter, Merck Life Science Kft., Hungary), followed by ultrafiltration and concentration via tangential flow filtration (TFF) using TFF-easy filters (HansaBioMed Life Sciences, Biocenter Laboratory Supplier Ltd., Budapest, Hungary). The EV-enriched supernatant was further purified by size exclusion chromatography (SEC) using qEVoriginal columns with 70 nm pore size (IZON, United States). Fractions 1 and 2 were collected and analyzed by nanoparticle tracking analysis (NTA) to determine the particle size and concentration of the isolated *Sm*_sEV.

### 2.3 Nanoparticle tracking analysis

The size distribution, median particle size, and concentration of *Sm*_sEV were determined by NTA using a ZetaView PMX-120 instrument (Particle Metrix GmbH, Meerbusch, Germany).

### 2.4 Surface-enhanced IR spectroscopy

Surface-enhanced infrared spectra were obtained using a Varian 2000 spectrometer (Scimitar Series, United States) equipped with a diamond attenuated total reflection (ATR) cell (“Golden Gate” single-reflection ATR unit, Specac, United Kingdom). Cysteamine-capped gold nanoparticles (AuNPs) were prepared following the protocol described in (Fuloria et al., 2021). Briefly, 22.36 mg (0.057 mmol) of HAuCl_4_.3H_2_O, 9.7 mg (0.085 mmol) of cysteamine hydrochloride (BioXtra, ≥98.1%, Sigma-Aldrich, Hungary), and 40 mL of Milli-Q (MQ) water were stirred at room temperature for 10 min. Then, 0.1 mL of freshly prepared 0.1 M sodium borohydride (NaBH_4_, ≥99.0%, Fluka, Hungary) solution was added, followed by 15 min of stirring at 700 rpm at room temperature. The freshly prepared AuNP solution was mixed with the EV sample in a 1:1 volume ratio. After 10 min of incubation, 3 µL of the mixture was pipetted onto the diamond ATR surface. The solvent was gently evaporated under ambient conditions for approximately 10 min to form a dry film. A total of 64 scans were collected at a spectral resolution of 2 cm^−1^. Data processing—including ATR correction, buffer background subtraction, and spectral analysis—was performed using the Grams/32 software package (Galactic Inc., United States).

### 2.5 Transmission electron microscopy

A 2 µL aliquot of the *Sm*_sEV sample was applied to a 200-mesh copper grid coated with formvar and allowed to adsorb for 2 min. Excess liquid was gently removed using filter paper. The grid was then floated on a drop of 2% (v/v) uranyl acetate for 1 min, blotted, and subsequently placed on a fresh drop of the same staining solution for an additional 1 min. After a final blotting step to remove excess stain, the grids were air-dried at room temperature. Imaging was performed using a FEI Morgagni 268D transmission electron microscope operated at an accelerating voltage of 80 kV.

### 2.6 Immunogold transmission electron microscopy

For immunogold transmission electron microscopy, we adapted previously published protocols with minor modification (Finamore et al., 2017; Wu et al., 2016). A 5 µL aliquot of EV suspension was applied to Formvar-coated nickel grids (SPI Supplies, United States) and incubated for 10 min at room temperature (RT). Excess liquid was removed, and EVs were fixed with 4% paraformaldehyde (PFA) for 10 min at RT, then rinsed three times with distilled water (5 min each). Grids were blocked with 2% sucrose in PBS (Molar Chemicals, Hungary) for 1 h, at RT. Primary antibody diluted in 2% sucrose/PBS was incubated overnight at 4 °C. After three 5-min washes with 2% sucrose, secondary antibody in 2% sucrose were applied for 1 h, at RT. The use of sucrose instead of BSA minimized non-specific binding and potential cross-reactivity. Unbound antibodies were removed with three 5-min sucrose washes followed by three PBS washes. Samples were post-fixed with 2% glutaraldehyde (Serva Electrophoresis GmbH, Germany) and rinsed three times with distilled water. Positive–negative staining was performed as described previously using UranyLess EM Stain (Electron Microscopy Sciences, United States). Visualization was done on a JEOL 1010 TEM (JEOL, Japan), and EV diameters were measured with ImageJ. For EV ESCRT marker ALIX detection, a rabbit polyclonal anti-ALIX (C-terminal) antibody (SAB4200477, Sigma-Aldrich, United States) and goat anti-mouse IgG conjugated to 10 nm gold particles (G7652, Sigma-Aldrich, United States) were used.

### 2.7 Labelling of EVs

To assess the *in vitro* internalization, *Sm*_sEV samples were labeled with the lipophilic fluorescent dye DiI (DiIC_18_; 1,1′-dioctadecyl-3,3,3′,3′-tetramethylindocarbocyanine perchlorate; Molecular Probes, United Kingdom) according to the manufacturer’s instructions. Briefly, 200 µL of EV sample was incubated with 2 µL of 1 mg/mL DiI for 60 min at 37 °C. Unbound dye was removed by SEC following a wash step with 400 µL of phosphate-buffered saline (PBS). As a negative control, the same volume of DiI dye was incubated in PBS (without EVs) under identical conditions, followed by washing and SEC, as described above.

### 2.8 Isolation and culture of primary human mesothelial cells and peritoneal fibroblasts

Primary human P-MCs were isolated from peritoneal biopsy tissue (SE RKEB: 199/2024) following a previously described protocol (Szebeni et al., 2024). Minced tissue was digested with 0.25% trypsin–EDTA at 37 °C for 30 min, washed with PBS, and centrifuged at 160 × g for 10 min. The pellet was seeded into 6-well plates and cultured in M199 medium containing 10% heat-inactivated FCS, hydrocortisone (400 nM), insulin (870 nM), HEPES (20 mM), EGF (3.3 nM), and 1% penicillin–streptomycin (all materials purchased from Life Technologies Kft., Hungary). Cells were maintained at 37 °C in 5% CO_2_ in collagen-coated flasks.

Peritoneal samples were collected during Tenckhoff catheter removal due to loss of ultrafiltration capacity (SE RKEB: 199/2024), as described previously (Szebeni et al., 2024). Primary human P-FBs were obtained by enzymatic digestion of peritoneal tissue with 1 mg/mL collagenase type II. The resulting suspension was cultured in DMEM/F-12 supplemented with 10% heat-inactivated FCS, 100 μg/mL streptomycin, and 100 U/mL penicillin (all materials purchased from Life Technologies Kft., Hungary) at 37 °C, in 5% CO_2_.

### 2.9 Cellular uptake of DiI-labeled EVs

P-MCs and P-FBs were seeded at a density of 6 × 10^4^ cells per well in a 4-well cell culture slide (Corning Costar, Sigma-Aldrich, Hungary) to reach approximately 80% confluence. Cells were then incubated with culture medium supplemented with 1 × 10^10^ particles of DiI-labeled EVs for 24 h at 37 °C in a humidified atmosphere containing 5% CO_2_. Following incubation, cell nuclei were stained with Hoechst 33342 (1:10,000 dilution; Merck Life Science Kft., Hungary) for 10 min at room temperature. After staining, the slides were mounted using ProLong™ Gold Antifade Mountant (Invitrogen, Hungary). Internalization of EVs was visualized using an Olympus IX-81 fluorescence microscope system (Olympus Corporation, Japan).

### 2.10 Cell proliferation assay (MTT)

P-MCs and P-FBs were seeded into 96-well plates at a density of 4 × 10^3^ cells/well. MTT assays were performed on 10 ng/mL PDGF-B (R&D Systems, United States) treated cells (n = 5 well/treatment group) in the absence or presence of 30 μL of 1 × 10^7^–1 × 10^10^ particles of *Sm*_sEV according to the instructions of the manufacturer (MTT Cell Proliferation/Viability Assay kit, R&D Systems, United States). Absorbance was recorded at 570 nm and at 690 nm as background in a SPECTROstar Nano microplate reader using SPECTROstar Nano MARS v3.32 software (BMG Labtech, Ortenberg, Germany). Vehicle-treated cells (4 mM hydrogen chloride (HCl) in case of PDGF, PBS in the case of EV) served as controls. Results were normalized and determined as the percentage ratio of control group values.

### 2.11 Collagen production assay (Sirius Red)

To evaluate fibrillar collagen content, a Sirius Red assay was performed (n = 5 wells per treatment group). P-FBs were treated with 10 ng/mL TGF-β1 for 48 h, with or without 30 μL 1 × 10^7^–1 × 10^10^ particles of *Sm*_sEV. Following treatment, cells were fixed for 15 min at room temperature in a solution containing 26% ethanol, 3.7% formaldehyde, and 2% glacial acetic acid. Samples were stained for 1 h at room temperature with 0.1% Sirius Red (Direct Red 80; Merck, Germany) dissolved in 1% acetic acid. After staining, wells were washed three times with 200 μL of 0.1 M HCl. The bound dye was then eluted with 100 μL of 0.1 M NaOH. Absorbance was measured at 544 nm, with 690 nm used as background correction, using a SPECTROstar Nano microplate reader and SPECTROstar Nano MARS v3.32 software (BMG Labtech, Germany). Vehicle-treated cells served as controls: 4 mM HCl for TGF-β1 and PBS for EV treatments. Results were normalized and expressed as a percentage of the corresponding control group.

### 2.12 Mouse model of peritoneal fibrosis and *in vivo* EV treatment

Animal experiments were conducted using 6–8-week-old male C57Bl/6J mice. Animals were housed in a temperature-controlled room (22 °C ± 1 °C) under a 12 h light/dark cycle with *ad libitum* access to standard rodent chow and water. All procedures were approved by the institutional animal care and use committee (approval number PE/EA/604-7/2020).

Peritoneal fibrosis was induced by daily intraperitoneal (i.p.) injections of 0.3 mL of 0.1% chlorhexidine digluconate (CG; Merck Life Science Kft, Budapest, Hungary) diluted in 15% ethanol and 85% phosphate-buffered saline (PBS) for seven consecutive days (CG group, n = 8). Control mice received daily i.p. injections of 0.3 mL PBS (Control group, n = 8).

A subset of CG- and PBS-treated animals received *Sm*_sEV (3 × 10^9^ particles in 0.3 mL PBS) via i.p. injection on days 1 and 4 (n = 8). At the end of the 7-day treatment period, mice were sacrificed, and parietal peritoneal tissue was collected. Peritoneal tissue samples were obtained from animals under anesthesia induced by an intraperitoneal injection of a ketamine–xylazine mixture (100 mg/kg ketamine and 10 mg/kg xylazine; total volume 100 µL). Samples were either fixed in 4% buffered formaldehyde for histological analysis or snap-frozen in liquid nitrogen and stored at −80 °C for molecular studies. Animals were subsequently euthanized by intraperitoneal administration of a ketamine–xylazine solution (300 mg/kg ketamine and 30 mg/kg xylazine; total volume 100 µL).

### 2.13 Masson’s trichrome staining

Formalin-fixed peritoneal tissues were embedded in paraffin and sectioned at a thickness of 3 μm. To evaluate peritoneal fibrosis, Masson’s trichrome staining was performed. Collagen-rich areas (stained blue) were quantitatively assessed using ImageJ software by outlining the perimeter of the positively stained regions. The average collagen-positive area per microscopic field was calculated and plotted. Additionally, submesothelial tissue thickness (in μm) was measured at ten independent points per section, and the average value was used for analysis.

### 2.14 Immunofluorescent staining

6 × 10^4^ P-MCs were seeded in 4-well cell culture chambers (Sarstedt, Germany) to reach approximately 80% confluence and were treated by 10 ng/mL TGF-β1 for 48 h, with or without 1 × 10^10^ particles of *Sm*_sEV. After fixation by methanol for 10 min at RT, chambers were first incubated with anti CK18 (sc 51582, Santa Cruz Biotechnology) and anti FN (AB2413, Abcam) primary antibodies for 1 h at RT; then, with the appropriate Alexa Fluor® conjugated secondary antibody (A10680, A21206, Life Technologies Kft., Hungary) for 60 min at RT. Nuclei were stained with Hoechst 33342 (Merck, Germany). Finally, the slides were cover-slipped with Invitrogen ProLong™ Gold Antifade Mountant (P36934, Life Technologies Kft., Hungary).

Peritoneal slides from the *in vivo* CG model were stained for COL1A1, FN and CK18. After fixation with Cytofix/Cytoperm (BD Cytofix/Cytoperm™ Fixation/Permeabilization Solution Kit, 554714, BD Pharmingen, San Diego, CA, United States), slides were incubated first with anti COL1A1-(sc293182, Santa Cruz Biotechnology) anti FN (AB2413, Abcam) or anti CK18 (sc 51582, Santa Cruz Biotechnology) primary antibodies (1 h, RT), followed by Alexa Fluor®-conjugated secondary antibodies (A21206, A21200, Life Technologies Kft., Hungary). DNA was stained with Hoechst. Finally, the slides were cover-slipped using Invitrogen ProLong™ Gold Antifade Mountant (P36934, Life Technologies Kft., Hungary). Appropriate negative controls were included by omitting the primary antibodies to ensure specific antibody binding and to control for autofluorescence.

### 2.15 Flow cytometry

Expression of heat shock protein (HSP), HSP70, HSP90 and Parkinson’s disease protein (PARK) 7 in *Sm*_sEVs was examined by CytoFlex nano (Beckman Coulter Inc., California, United States). To enable EV gating during FACS analysis, the *Sm*_sEVs were labeled with DiD membrane dye [DiIC18 solid (1,1′-Dioctadecyl-3,3,3′,3′-Tetramethylindodicarbocyanine, 4-Chlorobenzenesulfonate, Life Technologies Kft., Hungary] according to the manufacturer’s instructions. Unbound dye was removed by SEC following a wash step with PBS. Samples were then incubated with the anti-HSP70-(sc-1060, Santa Cruz Biotechnology), anti-HSP90 (C45G5, Cell Signaling), anti-PARK7 (ab18257, Abcam) and anti-HSP27 (NB110-57062, Novus Biologicals, United States) primary antibodies for 1 h/RT. After washing step with PBS and SEC the samples were incubated with the proper secondary antibodies for 1 h/RT (#A21206, #A21200, #A11055, Life Technologies Kft., Hungary).

### 2.16 Statistical analysis

Data were analyzed using GraphPad Prism 10.0 (GraphPad Software Inc., La Jolla, CA, United States). Normality was assessed with the Kolmogorov–Smirnov test, and statistical significance was evaluated using one-way ANOVA followed by Sidak’s multiple comparison test or by two-way ANOVA as appropriate. Results are presented as mean ± SD.

## 3 Results

### 3.1 Characterization of MACC-909 *Sm* strain and *Sm*_Sev

**FIGURE 1:**
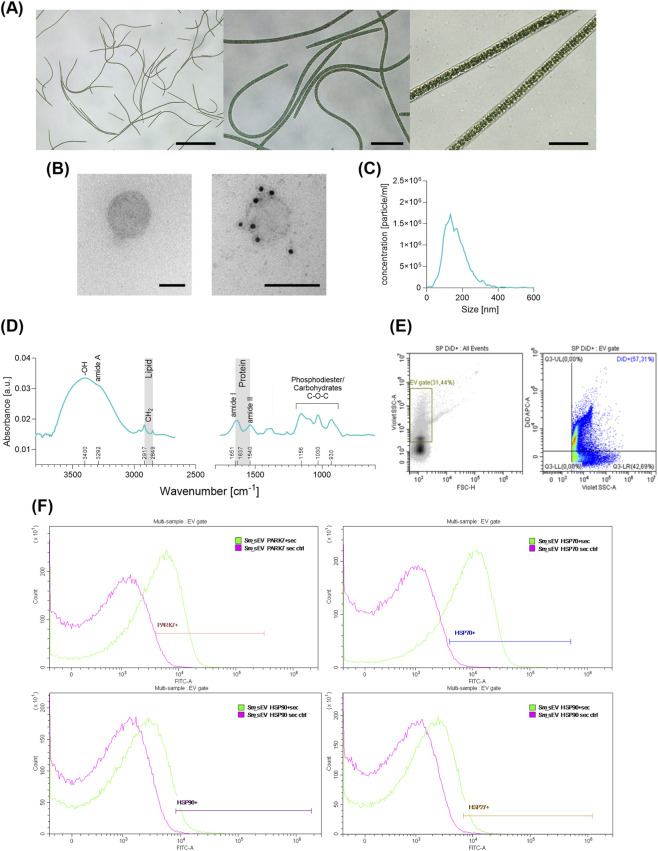
Characterization of *Spirulina maxima* and its derived extracellular vesicles (*Sm*_sEV). **(A)** Light microscopic observation of *Spirulina maxima*. Scale bar = 500, 100 and 35 μm respectively. **(B)** The morphology of *Sm*_sEV was visualized by transmission electron microscopy (TEM) and the presence of ALIX was confirmed by immunogold TEM using 10 nm gold particles. Scale bar = 100 nm. **(C)** The size distribution and particle concentration of *Sm*_sEV were determined using nanoparticle tracking analysis (NTA). **(D)** The protein and lipid contents and their ratio were analyzed based on the surface-enhanced infrared (SEIR) spectrum of the EV sample. **(E,F)** The expression of heat shock protein (HSP) 27, HSP70, HSP90, and Parkinson’s disease protein (PARK) 7 on the surface of DiD^+^ EVs (EV gate) was evaluated. Among these proteins, PARK7 and HSP70 were confirmed to be present.

The long, cylindrical filaments of the MACC-909 *Sm* strain were examined using light microscopy, while *Sm*_sEV were analyzed by electron microscopy to enable detailed visualization of their structures (Théry et al., 2006). (Figures 1A,B). TEM images revealed that *Sm*_sEV exhibited spherical morphology, and immunoelectron microscopy confirmed the presence of the ALIX protein on these vesicles, consistent with observations in other algae-derived EVs (Adamo et al., 2024) (Figure 1B). Nanoparticle tracking analysis (NTA) showed that the EVs had a mean hydrodynamic diameter of 150.0 ± 67.6 nm (mean ± SD) and a concentration of 1.8 × 10^11^ particles/mL (Figure 1C).

*Sm*_sEV samples were also analyzed by attenuated total reflection–Fourier-transform infrared spectroscopy (ATR-FTIR). To enhance the IR signature characteristic of EV components (lipids, proteins carbohydrates), the so-called surface-enhanced IR spectroscopy (SEIRS) was applied exploiting the unique plasmonic properties of gold nanoparticles (Bebesi et al., 2025). The SEIR spectrum obtained with home-designed cysteamine-capped gold nanoparticles (Bebesi et al., 2025; Veres-Székely et al., 2025) is presented in Figure 1D.

Contrary to normal IR spectrum, characteristic bands of proteins and lipids can be identified in the surface-enhanced IR spectrum. The bands at 3,292, 1,651/1,637 and 1,540 cm^−1^ correspond to amide A, amide I and amide II vibrations, respectively of the peptide backbone. There is a slight splitting of the amide I band: the shoulder at 1,651 cm^−1^ can be correlated to helical structures while the band at 1,637 cm^−1^ suggests the dominance of sheet-like forms. The presence of lipid is confirmed by the methylene stretchings of acyl chains of the phospholipids, at 2,917 and 2,849 cm^−1^.

From the area of amide I (fitted by a Gaussian function) and the C-H stretching region (integrated from 3,020 to 2,800 cm^−1^) a spectroscopic protein-to-lipid ratio of 2.27 ± 0.07 was calculated, characteristic to EV quality (Mihály et al., 2017; Théry et al., 2018). Our results are in line with our previous experiments on other EVs usually P/L_spectr_ falls between 0.5 and 2.5 (Veres-Székely et al., 2025; Kitka et al., 2019; Szentirmai et al., 2020; Szigyártó et al., 2018; Bebesi et al., 2022). Unlike human blood derived EVs, the fingerprint region of *Sm*_sEV sample is dominated by strong bands between 1,200 and 950 cm^−1^, assigned to C-O-C vibrations of carbohydrates, most likely derived from intracellular, extracellular and cell wall saccharides, commonly synthesized by macroalgal and microalgal systems (Sarkar et al., 2024).

Using a gate validated with *Sm*_sEVs DiD staining (Figure 1E), we assessed the expression levels of HSP27, HSP70, HSP90, and PARK7 in *Sm*_sEVs. PARK7 and HSP70 were clearly positive relative to the secondary control, HSP90 showed only a weak signal, and HSP27 did not exceed the secondary control (Figures 1E,F).

### 3.2 Internalization of *Sm*_sEV

**FIGURE 2:**
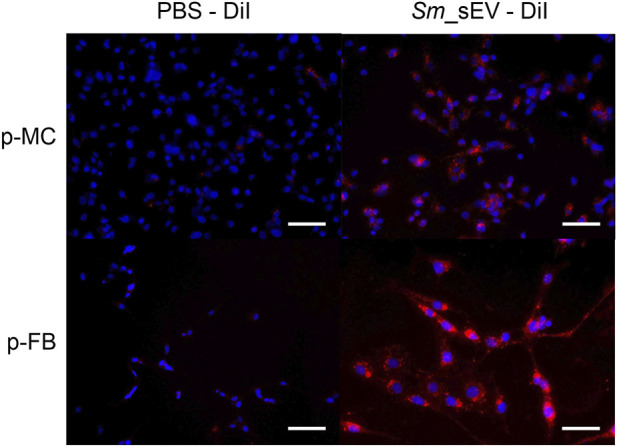
Internalization of DiI-labeled *Spirulina maxima* extracellular vesicles (*Sm*_sEV) into human primary peritoneal mesothelial (p-MC) and fibroblast cells (p-FB). Cells were incubated with DiI-labeled *Sm*_sEV for 24 h, after which internalization was examined using fluorescence microscopy. Cell nuclei were counterstained with Hoechst 33342 (blue). Scale bar = 100 μm.

P-MCs displayed the characteristic cobblestone morphology and were positive for CK-18 (Kitka et al., 2019). P-FBs were identified based on their characteristic spindle-like morphology and positive expression of α-SMA, fibronectin, and vimentin confirming their fibroblastic identity as previously described (Szebeni et al., 2024). The DiI-labeled *Sm*_sEV were internalized by P-MCs and P-FBs to a significant extent (Figure 2).

### 3.3 Effect of *Sm*_sEV on TGF-β and PDGF-B-induced activation of P-MCs

**FIGURE 3:**
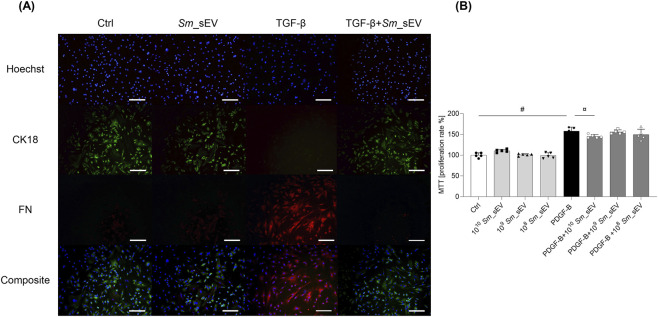
Effect of *Spirulina maxima* extracellular vesicles (*Sm*_sEV) on the mesenchymal transition and proliferation of peritoneal mesothelial cells. **(A)** Primary peritoneal mesothelial cells were stimulated by TGF-β 10 ng/mL in the presence or absence of 1 × 10^10^ *Sm*_sEV. The presence of CK-18 and FN was investigated by immunofluorescent staining. Scale bar = 200 μm. **(B)** Primary human peritoneal mesothelial cells were stimulated with 10 ng/mL PDGF-B in the presence or absence of *Sm*_sEV at various concentrations (1 × 10^8^–1 × 10^10^ particles) for 24 h. Cell proliferation was assessed using the MTT assay. Data are presented as mean ± SD (n = 5) and analyzed using one-way ANOVA followed by Sidak’s multiple comparison test. Significance levels are indicated on the figure, (#) p ≤ 0.0001 Ctrl vs. PDGF-B, (¤) p = 0.0462 PDGF-B vs. PDGF-B+10^10^ *Sm*_sEV.

Under control conditions, P-MCs displayed strong positive staining for the epithelial marker cytokeratin-18 (CK-18), while fibronectin (FN) staining was absent, consistent with our previous findings (Szebeni et al., 2024). Following 48 h of TGF-β treatment, cells became strongly FN-positive and lost their CK-18 staining, indicating progression toward a fibrotic phenotype. Notably, treatment with *Sm*_sEV restored the CK-18 positivity of TGF-β treated P-MCs (Figure 3A). In addition, *Sm*_sEV treatment reduced PDGF-B-induced P-MC proliferation in a dose-dependent manner (Figure 3B).

### 3.4 Effect of *Sm*_sEV on PDGF-B-induced proliferation and TGF-β-induced collagen accumulation of P-FBs

**FIGURE 4:**
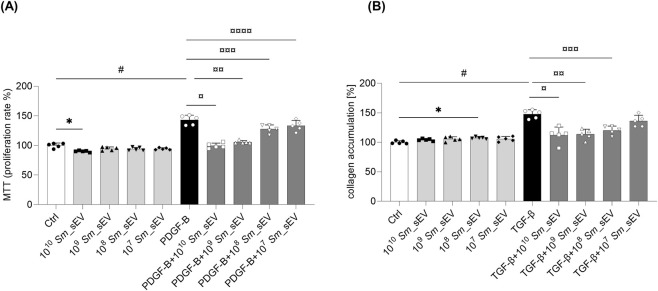
Effect of *Spirulina maxima* extracellular vesicles (*Sm*_sEV) on the proliferation and collagen accumulation of human peritoneal fibroblast cells. **(A)** Primary human peritoneal fibroblast cells were stimulated with 10 ng/mL PDGF-B in the absence or presence of *Sm*_sEV at various concentrations (1 × 10^7^–1 × 10^10^ particles) for 24 h. Cell proliferation was assessed using the MTT assay. Data are expressed as mean ± SD (n = 5) and analyzed using one-way ANOVA followed by Sidak’s multiple comparison test. Significance levels are indicated on the figure, (*) p = 0.0310 Ctrl vs.10^10^ *Sm*_sEV, (#) p ≤ 0.0001 Ctrl vs. PDGF-B, (¤) p ≤ 0.0001 PDGF-B vs. PDGF-B+10^10^ *Sm*_sEV, (¤¤) p ≤ 0.0001 PDGF-B vs. PDGF-B+10^9^ *Sm*_sEV, (¤¤¤) p = 0.0002 PDGF-B vs. PDGF-B+10^8^ *Sm*_sEV, (¤¤¤¤) p = 0.0363 PDGF-B vs. PDGF-B+10^7^ *Sm*_sEV. **(B)** Primary human peritoneal fibroblast cells were stimulated with 10 ng/mL TGF-β in the absence or presence of *Sm*_sEV at various concentrations (1 × 10^7^–1 × 10^10^ particles) for 48 h. Collagen production was assessed using the Sirius Red assay. Data are presented as mean ± SD (n = 5) and analyzed by one-way ANOVA followed by Sidak’s multiple comparison test. Significance levels are indicated on the figure (*) p = 0.0065 Ctrl vs.10^8^ *Sm*_sEV, (#) p = 0.0002 Ctrl vs. TGF-β, (¤) p = 0.0148 TGF-β vs. TGF-β +10^10^ *Sm*_sEV, (¤¤) p = 0.0011 TGF-β vs. TGF-β +10^9^ *Sm*_sEV, (¤¤¤) p = 0.0026 TGF-β vs. TGF-β +10^8^ *Sm*_sEV.

*Sm*_sEV treatment dose-dependently reduced the PDGF-B-induced proliferation of P-FBs (Figure 4A). Furthermore, *Sm*_sEV treatment dose-dependently decreased the TGF-β-induced collagen accumulation in P-FBs (Figure 4B).

### 3.5 Effect of *Sm*_EVs on peritoneal scar tissue formation *in vivo*

**FIGURE 5:**
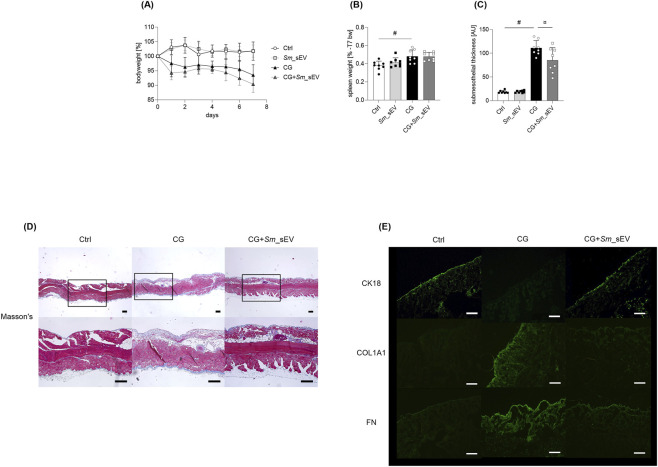
Effect of *Spirulina maxima* extracellular vesicles (*Sm*_sEV) on peritoneal fibrosis. Mice received daily intraperitoneal (i.p.) injections of chlorhexidine digluconate (CG) for 7 days, with or without *Sm*_sEV (3 × 10^9^ particles in 0.3 mL PBS) co-treatment administered on days 1 and 4. Body weight loss and spleen weight were measured to assess the safety of *Sm*_sEV **(A,B)**. There was no significant difference in body weight loss among all investigated groups (two-way ANOVA). The significance level is indicated on Panel **(B)** (#) p = 0.0150 Ctrl vs. CG. Data are expressed as mean ± SD (n = 8) and analyzed by one-way ANOVA followed by Sidak’s multiple comparison test. Submesothelial thickness was examined in Masson’s trichrome–stained peritoneal sections **(D)** and quantified by graphical analysis **(C)**. Scale bar = 200 μm. Data are presented as mean ± SD (n = 8) and analyzed using one-way ANOVA followed by Sidak’s multiple comparison test. Significance levels are indicated on the figure (#) p ≤ 0.0001 Ctrl vs. CG, (¤) p = 0.0066 CG vs. CG + *Sm*_sEV. The presence of cytokeratin-18 (CK-18), fibronectin (FN), and collagen type I (COL1A1) were evaluated by immunofluorescence staining of parietal peritoneal sections. Scale bar = 200 μm **(E)**. AU, arbitrary unit.

To investigate the *in vivo* effects of *Sm*_sEV, a well-established CG-induced mouse model of peritoneal fibrosis was employed, as previously described (Szebeni et al., 2024). In control mice, the peritoneum showed positive staining for the epithelial marker cytokeratin-18 (CK-18), with little to no expression of fibrosis markers such as fibronectin (FN) and collagen type I alpha (COL1A1) (Figure 5E). In contrast, strong presence of FN and COL1A1, along with the absence of CK-18 staining, was observed in the peritoneum of CG-treated mice, indicating fibrotic transformation of the peritoneal mesothelial cells. Treatment with *Sm*_sEV markedly inhibited the CG-induced immunohistochemical changes, suppressing FN and Col1-α expression while restoring CK-18 positivity (Figure 5E).

These molecular findings were consistent with histological observations: CG treatment caused a pronounced thickening of the submesothelial zone—from 18.55 μm in control mice to 111.23 μm—which was significantly reduced following *Sm*_sEV administration (to 85.10 μm) (Figures 5C,D). Importantly, *Sm*_sEV treatment did not result in body weight loss compared with either control or CG-treated groups. In addition, spleen weight remained unchanged, suggesting that the administered dose of *Sm*_sEV did not elicit a systemic inflammatory response (Figures 5A,B).

## 4 Discussion

In the present study, we investigated the role of *Spirulina maxima*-derived EVs (*Sm*_sEV) in PD related peritoneal fibrosis. Peritoneal fibrosis is characterized by progressive thickening of the peritoneal membrane resulting from excessive ECM accumulation, leading to impaired ultrafiltration capacity. In severe cases, life-threatening encapsulating peritoneal sclerosis (EPS) may occur in 0.5%–4.4% of patients undergoing PD. EPS is a critical complication that leads to death in 50% of the cases within 12 months. Despite the severity of EPS, similarly to other fibroproliferative diseases, there is still no drug available to cure it.

Previously, we and others have shown that various stem cell-derived EVs (MSC-EV) significantly inhibit experimental peritoneal fibrosis (Szebeni et al., 2024; Gouveia et al., 2025; Zhao et al., 2024; Yu et al., 2023; Li et al., 2013). Although the mechanism is still not clear it is widely accepted that MSC-EV have strong regenerative potential (Fuloria et al., 2021; Yin et al., 2020; Rezabakhsh et al., 2021; Keshtkar et al., 2018). Although MSC-EV-based therapy holds great promise as a novel “cell-free” therapeutic approach, several critical issues remain to be overcome before its clinical application (Fuloria et al., 2021).

Indeed, since culture conditions can alter the behavior of MSCs and the quality of the EVs they release, standardized culture protocols are needed to ensure consistency across different production batches. Also, the lifespan of MSCs - and therefore the number of EVs they release - is limited, making the production of a therapeutic dose of EVs challenging. Although several other issues remain to be solved, we would like to highlight one more: the cargo of MSC-EVs may contain unwanted proteins, RNAs, or other biomolecules that could cause side effects.

Some of these problems can be addressed by using plant- and especially algae-derived EVs, which can be produced in large suspension batches, ensuring batch-to-batch consistency of the resulting vesicles. Algae constitute an integral part of the human diet, primarily as food additives, without significant allergic or toxic side effects. Therefore, it is not surprisingly, that recent publications support the notion that certain algal EVs exhibit good biocompatibility and low toxicity in mammalian systems (Garaeva et al., 2025; Adamo et al., 2024; Brizzi et al., 2025). Accordingly, in recent years, interest in algae-derived EVs has exponentially increased as a scalable and sustainable alternative to EVs derived from mammalian sources (Lian et al., 2022; Feng et al., 2023; Karamanidou and Tsouknidas, 2021; Mu et al., 2023; Bai et al., 2024; López d et al., 2023). However, algae represent a large group of living organisms, and some produce potent toxins, including hepatotoxins such as microcystins and neurotoxins such as saxitoxins, while others live in symbiosis with prokaryotes; therefore, care should be taken when selecting the appropriate strain for medical use (Villalobos et al., 2025).

Therefore, in the present study, we investigated the effects of EVs derived from the well-known food alga, the *Sm* (Figure 1A) on peritoneal scarring.

First the investigated nanoparticles isolated from the medium of *Sm* were characterized. Indeed, *Sm*_sEV showed spherical morphology, a hydrodynamic diameter and protein-to-lipid ratio typical of EVs, and expressed the EV marker ALIX (Figures 1B–D). Although these EVs were of algal origin, we found that in accordance with previous studies (Garaeva et al., 2025; Adamo et al., 2024), they were able to penetrate human primary P-MCs and P-FBs (Figure 2).

TGF-β and PDGF-B are well-known profibrotic factors, whose roles have been demonstrated in various fibroproliferative diseases (Ying et al., 2017; Zhou et al., 2024; Biernacka et al., 2011; Samarakoon et al., 2013; Trojanowska, 2008), including peritoneal scarring (Szebeni et al., 2024; Lai et al., 1999). During PD, the released profibrotic factors directly interact with the peritoneal mesothelial cells of the peritoneum (Szebeni et al., 2024; Lai et al., 1999; Zweers et al., 1999), and these cells can transform into fibroblast-like mesenchymal cells through a process called epithelial–mesenchymal transition (EMT) (Figure 3A). TGF-β plays a central role in this process. Therefore, in the first set of experiments we investigated the effect of the *Sm*_sEV on the TGF-β-induced EMT in primary human P-MCs. Consistent with our previous findings we observed that TGF-β treatment reduced CK-18 and increased FN immunopositivity in P-MCs, indicating their mesenchymal transition (Szebeni et al., 2024). Interestingly, we found that *Sm*_sEVs inhibited this process - the TGF-β-induced EMT in P-MCs (Figure 3A).

Similarly, we found that *Sm*_sEVs inhibited PDGF-B-induced proliferation in mesothelial cells (Figure 3B), further strengthening our observations regarding the effect of the *Sm*_EVs on EMT in P-MCs.

In the next series of experiments, we investigated the effect of *Sm*_sEVs on PDGF-B-induced proliferation and TGF-β-induced collagen production in human primary P-FBs. Similar to P-MCs, we found that *Sm*_sEVs dose dependently inhibited both PDGF-B-induced proliferation and TGF-β-induced collagen production in these cells, suggesting a marked antifibrotic effect of the *Sm*_sEVs (Figures 4A,B).

Since *Sm*_sEV significantly inhibit both TGF-β or PDGF-B-induced fibrotic processes, which are common in various fibroproliferative diseases, our data suggest that *Sm*_sEV may contribute to the development of new strategies for treating not only peritoneal fibrosis but also other fibroproliferative diseases.

Moreover, to our surprise, all of the above observations were consistent with our previous study investigating the effect of human EVs derived from peritoneal dialysate (PDE EV) (Szebeni et al., 2024), suggesting the presence of a conserved mechanism of action that enables EVs of both human and algal origin to inhibit PDGF-B- and TGF-β driven profibrotic pathways. Indeed, several conserved molecules, including HSP27, HSP70, HSP90, PARK7 have been reported to inhibit PDGF-B and/or TGF-β-driven pathways (Hirano et al., 2004; Li et al., 2011; Taipale et al., 2010; Yin et al., 2021). Therefore, we assessed whether Sm_sEV contains one or more of these conserved chaperones within their cargo.

We found that *Sm*_sEVs were markedly positive for PARK7 and HSP70 (Figure 1F), both of which effectively suppress inflammation (Yin et al., 2021; Zhang et al., 2022; Lippai et al., 2021) and epithelial–mesenchymal transition (Li et al., 2011) the critical drivers of fibrosis (Yin et al., 2021; Lippai et al., 2021; Tanaka et al., 2010). Moreover, Li and coworkers demonstrated that of HSP70 prevents receptor-dependent phosphorylation and nuclear translocation of Smad2 and blocks TGF-β–induced epithelial–mesenchymal transition (EMT) in HaCaT cells (Li et al., 2011). It is also noteworthy that HSP70 was also present in the cargo of the PDE-EVs we previously studied (Szebeni et al., 2024), which also exhibited strong antifibrotic properties. Similarly, we previously demonstrated that PARK7 decreases the expression of the well-known profibrotic factor TGF-β in the colon of mice with DSS-induced colitis *in vivo* (Lippai et al., 2021), and Gao et al. (2017) reported that PARK7 suppresses the TGF-β/Smad signaling pathway. These observations suggest that certain evolutionarily conserved cargo components, such as chaperones, may contribute, at least in part, to the antifibrotic effects of algal EVs. However, further studies using genetically engineered algal cells or algal EVs loaded with PARK7 or HSP70 are needed to establish the causal relationship between the investigated chaperone and fibrosis.

Finally, a major question remains as to whether the observed effect is sufficiently strong to inhibit fibrosis *in vivo*. Therefore, we investigated the effect of *Sm*_sEV in a CG-induced mouse model of peritoneal fibrosis. This model is particularly well-suited for investigating the role of EVs in fibrosis, as the site of EV administration coincides with the site of scarring, eliminating potential issues related to EV absorption or clearance.

In accordance with our previous study using human EVs of peritoneal dialysate origin (Szebeni et al., 2024), we observed that treatment with cyanobacteria-derived *Sm*_sEV on the first and fourth days of CG administration significantly inhibited the submesothelial thickening of the peritoneum (Figures 5C,D).

We found that the CG-induced submesothelial thickening, as demonstrated by Masson’s trichrome staining of peritoneum samples (Figures 5C,D), and–consistent with our *in vitro* results - the decreased mesothelial CK18 together with the increased submesothelial COL1A1 and FN immunopositivity were all diminished by *Sm*_sEV treatment of the mice (Figure 5E), suggesting the strong *in vivo* antifibrotic effect of *Sm*_sEV.

Furthermore, the administered dose of *Sm*_sEV did not elicit a systemic inflammatory response and was well tolerated, no behavioral changes were observed (Figures 5A,B). However, future studies with larger cohorts, as well as lower and/or more frequently administered doses of Sm-sEVs could help determine the minimum therapeutic dose and better explore the therapeutic relevance of this type of EV. Our results is in line with the previous studies suggesting the excellent biocompatibility of algal EVs (Garaeva et al., 2025; Adamo et al., 2024; Conlon and Touzet, 2025). Moreover, given the similarity of fibrotic mechanisms across different organs, these results suggest that the effects of *Sm*_sEV may extend beyond the peritoneum to other fibroproliferative diseases.

In summary, we demonstrated that EVs derived from the cyanobacterium *S. maxima* are capable of inhibiting profibrotic TGF-β and PDGF-B driven fibroblast activation *in vitro*, as well as CG-induced peritoneal fibrosis *in vivo*. Our observations further support the notion that EVs of different origins may be suitable for mitigating the detrimental effects of fibrosis. However, further studies are needed to clarify the underlying mechanisms. Our present observations, together with our previous study using human PDE EVs, suggest the existence of a common antifibrotic mechanism of EVs of different origins.

## Data Availability

The original contributions presented in the study are included in the article/supplementary material, further inquiries can be directed to the corresponding author.
